# Hybrid One- and Two-sided Flow-Encodings Only (HOTFEO) to accelerate 4D flow MRI

**DOI:** 10.1186/1532-429X-18-S1-P364

**Published:** 2016-01-27

**Authors:** Da Wang, Jiaxin Shao, Daniel B Ennis, Peng Hu

**Affiliations:** 1 grid.19006.3e0000000096326718Department of Radiological Sciences, David Geffen School of Medicine, University of California, Los Angeles, CA USA; 2 grid.19006.3e0000000096326718Biomedical Physics Interdepartmental Graduate Program, University of California, Los Angeles, CA USA

## Background

**Figure 1:**
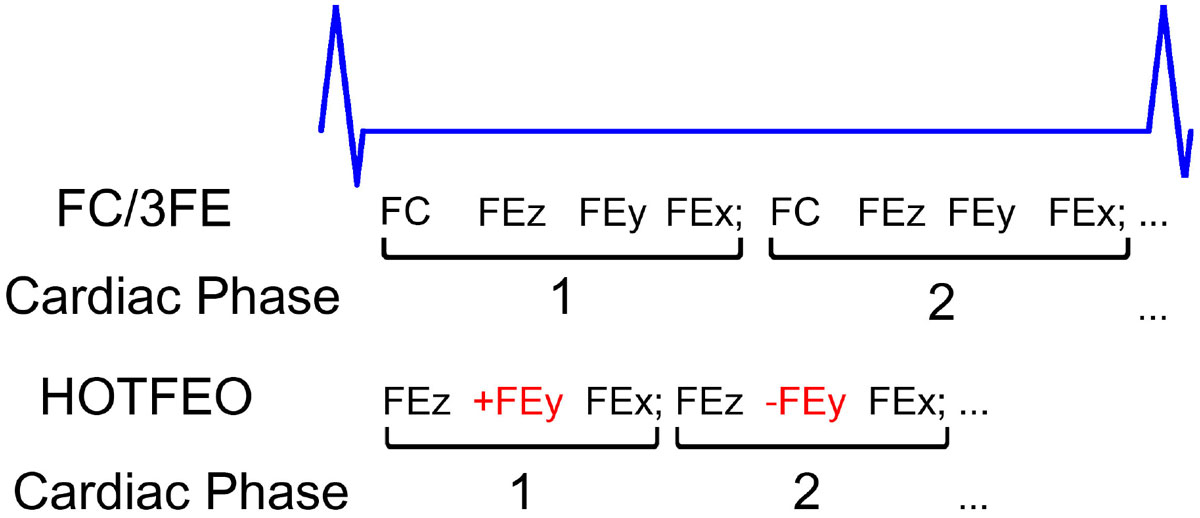
**The acquisition strategies of reference FC/3FE and HOTFEO: the FC/3FE acquired four echoes to generate each cardiac phase**. The HOTFEO used only three acquisitions (two-sided FE in y-direction and one-sided FE in x/z-direction) to generate each cardiac phase.

4D flow phase-contrast MRI (PC-MRI) has been extensively used for visualization and quantification of blood flow and velocity. It typically acquires one flow-compensated (FC) and three-directional (3D) flow-encoded (FE) echoes (FC/3FE) to update one cardiac phase, which often limits the achievable temporal resolution and temporal footprint for each cardiac phase. In this work, we propose a Hybrid One- and Two-sided Flow-Encodes Only (HOTFEO) acquisition strategy (as shown in Figure 1) that incorporates with a velocity direction constraint (assuming the velocity direction, not magnitude, changes very little between two cardiac phases) to accurately calculate without acquiring FC data to achieve 4/3-fold acceleration. Retrospective and prospective in vivo studies were performed to validate the measurement accuracy of total volumetric flow and maximal total peak velocity.

## Methods

In many vascular territories, such as common carotids arteries (CCAs) and circle of Willis, the blood flow tends to be laminar flow and the velocity direction and the FC signal phase does not change significantly between two cardiac phases (~140 ms). In our PC-MRI sequence shown in Figure 1, we only acquire the 3D FE data except that the phase-encoding FE acquisition is interleaved two-sided FE. Thus, the velocity direction constraint for cardiac phase n and n+1 for calculating FC phase ϕ_FCn_(= ϕ_FCn+1_) is:

ϕ_FCn_=argmin _ϕFCn_ ||**V**_n_*V_**n+1**_|-|**V**_**n**_|*|**V**_**n+1**_|| [1]

**V**_n_*V_**n+1**_ is the dot product of two velocity vectors that contains 3D velocity information: V_n,x/y/z_=(ϕ_FEn,x/y/z_ - ϕ_FCn_)/π*VENC; ϕ_FEn,x/y/z_ is the acquired FE phase signal in the x/y/z direction for cardiac phase n, |**V**_**n**_| is the velocity magnitude for cardiac phase n. Eq. [1] essentially minimizes the angle between the velocity directions between two adjacent cardiac phases.

Six healthy volunteers were recruited for retrospective (using standard reference 4D flow data to simulate the HOTFEO acquisition) and prospective *in vivo* study using a 3T scanner (Skyra, Siemens) with a 4-channel neck coil, using both standard 4D flow and the proposed HOTFEO sequence. Both sequences were implemented with VENC = 100-105 cm/s, flip angle = 20°, readout bandwidth = 815 Hz/Pixel, TE = 3.35 ms, Views-per-segment = 3(FC/3FE) and 4(HOTFEO), temporal resolution = 68 ms, acquired matrix = 256 × 176 × 10, FOV = 256 × 176 × 18.2 mm^3^. All scans were acquired during free breathing with prospective ECG gating.

## Results

**Figure 2:**
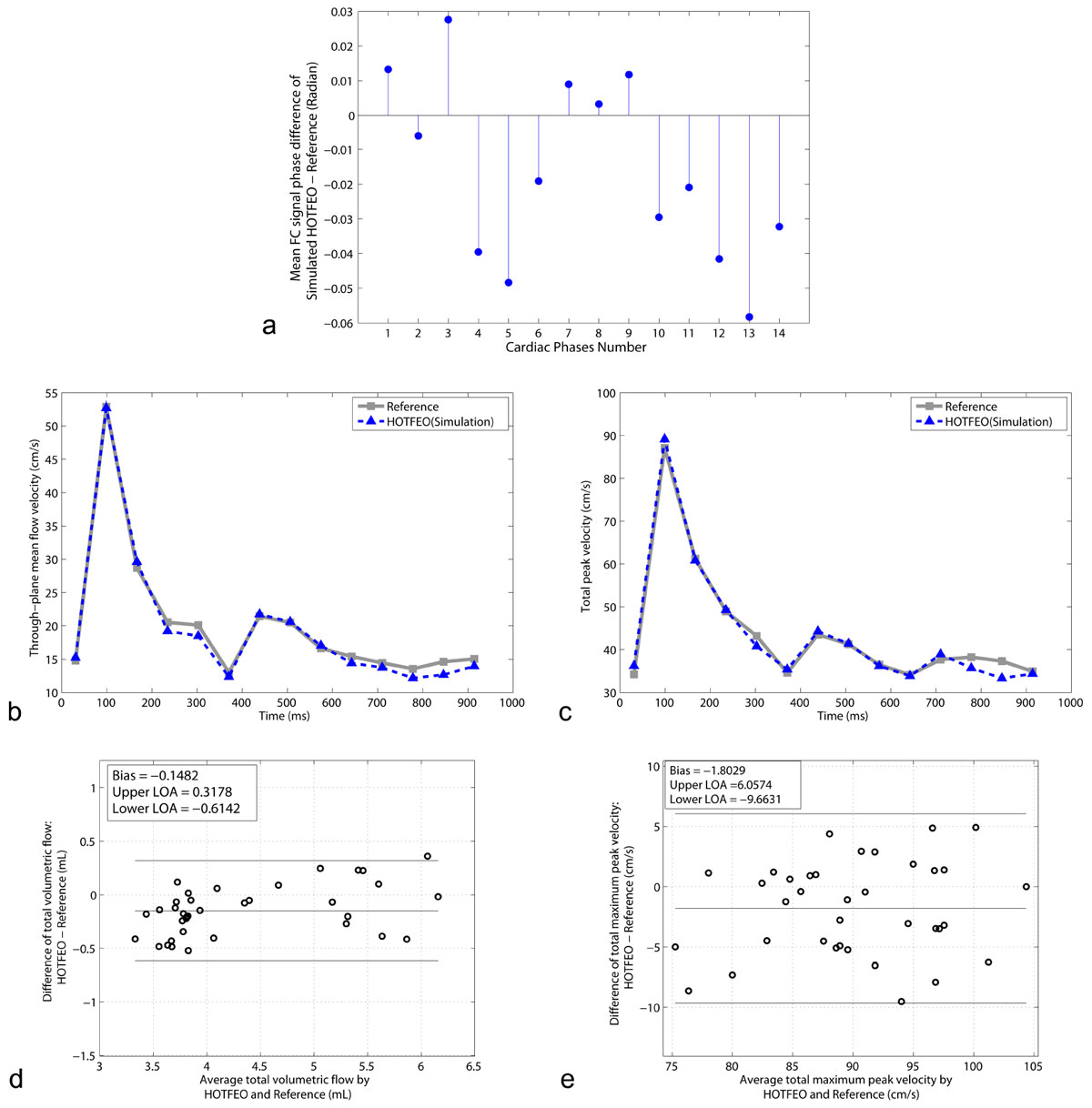
**a. An example of phase differences between the background phase calculated using simulated HOTFEO and the actual measured FC data**. b. The through-plane mean flow velocity waveforms of the reference FC/3FE PC-MRI (gray) and simulated HOTFEO (blue). c. The total peak velocity waveforms of the reference FC/3FE PC-MRI (gray) and simulated HOTFEO (blue). The HOTFEO technique provides accurate background phase estimation without acquiring the FC data, and provides accurate flow velocity measurements. d. total volumetric flow measurement between prospective HOTFEO and reference 4D flow. The bias is -0.1 mL (-3.4% relative bias error) and the interval between the upper and lower limits of agreement was [-0.6, 0.3] mL. e. The Bland-Altman plots of total maximum peak velocity measurement between prospective HOTFEO and reference 4D flow. The bias is -1.8 cm/s (2.0% relative bias error) and the interval between the upper and lower limits of agreement was [-9.7, 6.1] cm/s.

Compared with standard 4D flow, simulated HOTFEO showed that the FC calculation (Figure 2a) is accurate with mean RMSE = 0.04(range:0.02-0.06) rad and velocity waveforms (Figure 2bc) have a good agreement. Bland-Altman tests showed that prospective 4/3-fold accelerated HOTFEO technique resulted in relatively small bias errors and good agreements for total volumetric flow (-3.4%), and total maximum peak velocity (-2.0%) measurements in CCAs (Figure 2de).

## Conclusions

HOTFEO can accelerate 4D flow PC-MRI while maintaining the measurement accuracy of total volumetric flow and total maximum peak velocity measurements.

